# Comparative Effectiveness of a Web-Based Patient Decision Aid for Therapeutic Options for Sickle Cell Disease: Randomized Controlled Trial

**DOI:** 10.2196/14462

**Published:** 2019-12-04

**Authors:** Lakshmanan Krishnamurti, Diana Ross, Cynthia Sinha, Traci Leong, Namita Bakshi, Nonita Mittal, Divya Veludhandi, Anh-Phuong Pham, Alankrita Taneja, Kamesh Gupta, Julum Nwanze, Andrea Marie Matthews, Saumya Joshi, Veronica Vazquez Olivieri, Santhi Arjunan, Ifechi Okonkwo, Ines Lukombo, Peter Lane, Nitya Bakshi, George Loewenstein

**Affiliations:** 1 Aflac Cancer and Blood Disorders Center Emory University School of Medicine Children’s Healthcare of Atlanta Atlanta, GA United States; 2 Department of Biostatistics and Bioinformatics Rollins School of Public Health Emory University Atlanta, GA United States; 3 Center for Behavioral Decision Research Department of Social and Decision Sciences Carnegie Mellon University Pittsburgh, PA United States

**Keywords:** decision aids, decision support, sickle cell anemia, sickle cell disease, sickle cell disorders

## Abstract

**Background:**

Hydroxyurea, chronic blood transfusions, and bone marrow transplantation are efficacious, disease-modifying therapies for sickle cell disease but involve complex risk-benefit trade-offs and decisional dilemma compounded by the lack of comparative studies. A patient decision aid can inform patients about their treatment options, the associated risks and benefits, help them clarify their values, and allow them to participate in medical decision making.

**Objective:**

The objective of this study was to develop a literacy-sensitive Web-based patient decision aid based on the Ottawa decision support framework, and through a randomized clinical trial estimate the effectiveness of the patient decision aid in improving patient knowledge and their involvement in decision making.

**Methods:**

We conducted population decisional needs assessments in a nationwide sample of patients, caregivers, community advocates, policy makers, and health care providers using qualitative interviews to identify decisional conflict, knowledge and expectations, values, support and resources, decision types, timing, stages and learning, and personal clinical characteristics. Interview transcripts were coded using QSR NVivo 10. Alpha testing of the patient decision aid prototype was done to establish usability and the accuracy of the information it conveyed, and then was followed by iterative cycles of beta testing. We conducted a randomized clinical trial of adults and of caregivers of pediatric patients to evaluate the efficacy of the patient decision aid.

**Results:**

In a decisional needs assessment, 223 stakeholders described their preferences, helping to guide the development of the patient decision aid, which then underwent alpha testing by 30 patients and 38 health care providers and iterative cycles of beta testing by 87 stakeholders. In a randomized clinical trial, 120 participants were assigned to either the patient decision aid or standard care (SC) arm. Qualitative interviews revealed high levels of usability, acceptability, and utility of the patient decision aid in education, values clarification, and preparation for decision making. On the acceptability survey, 72% (86/120) of participants rated the patient decision aid as good or excellent. Participants on the patient decision aid arm compared to the SC arm demonstrated a statistically significant improvement in decisional self-efficacy (*P*=.05) and a reduction in the informed sub-score of decisional conflict (*P*=.003) at 3 months, with an improvement in preparation for decision making (*P*<.001) at 6 months. However, there was no improvement in terms of the change in knowledge, the total or other domain scores of decisional conflicts, or decisional self-efficacies at 6 months. The large amount of missing data from survey completion limited our ability to draw conclusions about the effectiveness of the patient decision aid. The patient decision aid met 61 of 62 benchmarks of the international patient decision aid collaboration standards for content, development process, and efficacy.

**Conclusions:**

We have developed a patient decision aid for sickle cell disease with extensive input from stakeholders and in a randomized clinical trial demonstrated its acceptability and utility in education and decision making. We were unable to demonstrate its effectiveness in improving patient knowledge and involvement in decision making.

**Trial Registration:**

ClinicalTrials.gov NCT03224429; https://clinicaltrials.gov/ct2/show/NCT03224429 and ClinicalTrials.gov NCT02326597; https://clinicaltrials.gov/ct2/show/NCT02326597

## Introduction

Sickle cell disease (SCD) is a major public health problem in the United States, affecting an estimated 100,000 individuals [1] and associated with significant morbidity caused by pain crises, acute chest syndrome, stroke, pulmonary hypertension, leg ulcers, and irreversible organ damage [2]. SCD has substantial health care utilization, with total annual charges in the United States that exceed $1.5 billion [3,4]. Quality of life is impaired [5-12], productivity is diminished (with a 40-60% unemployment rate [13]), and there is increased risk of premature mortality, especially in patients with the genotypes HbSS or HbS/β° thalassemia [2,14-18]. SCD predominantly affects ethnic minorities, with African Americans, followed by Hispanics, being the largest affected communities [18]. Disease-modifying therapies, such as hydroxyurea (HU), chronic transfusion therapy (CTT), and bone marrow transplantation (BMT), have demonstrated efficacy in clinical trials. HU is effective at reducing complications and health care utilization in children and adults, and improves survival in adults [19-25]; however, it must be taken daily and indefinitely with regular monitoring for side effects, and its impact on long-term organ function is unknown. HU is underprescribed and when prescribed is underutilized, with 85% of SCD patients who received a HU prescription never filling it [19,23,25,26] and an average prescription refill rate of 58% [27]. Over 20% of families refuse HU, citing reasons such as fear of cancer or other side effects, concern about lack of efficacy, and unwillingness to take the medicine or make the additional visits to clinic or pharmacy [23,26]. BMT is potentially curative, but is associated with treatment-related morbidity, risk of mortality, and later effects such as infertility [28]. Only a small minority of eligible patients undergo BMT even when an HLA-identical sibling donor is available [29,30]. CTT is efficacious in primary and secondary prevention of stroke but is associated with significant risks such as allo-immunization and iron overload [31-34]. In making choices regarding HU, BMT, or CTT, patients and caregivers are most influenced by perceived efficacy and safety [35,36]. There are trade-offs between the benefits and harms between the different treatment options, such that an individual patient’s preference, values, and risks of different outcomes could influence their decisions. A major contributor to decisional dilemma associated with treatments for SCD is the absence of studies to compare the benefits and harms of these treatments and guide patients in their choice of treatments. Thus, there is a need for research that helps to understand patient values and preferences to determine how to help them make informed treatment decisions in line with these values and preferences.

The Ottawa Decision Support Framework is an evidence-based, practical guideline for assisting patients in making health or social decisions [37-40]. It uses a three-step process to: (1) assess client and practitioner determinants of decisions to identify decision support needs; (2) provide decision support through counseling and decision tools; and (3) evaluate decision making. The Ottawa decisional support framework has been used to guide the development and evaluation of more than 30 patient decision aids, practitioner decision support resources, and tools to evaluate the quality and outcomes of decisions [37-40].

The overarching objective of this study was to develop a Web-based patient decision aid, drawing input from patients, their caregivers, and other stakeholders to meet the decisional needs of patients with SCD considering various treatment options, and then to test the efficacy of the patient decision aid in real life conditions. The research question was whether a Web-based decision aid that is comprehensible, acceptable, and usable, and would meet the decisional needs of those with SCD, is feasible. We hypothesized that the use of a patient decision aid would help patients and families better navigate treatment choices. Further, we proposed that a Web-based patient decision aid would help clarify patient values for themselves and for their health care providers. 

## Methods

### Needs Assessment

We identified and recruited stakeholders connected with decision making for SCD. We included: (1) individuals with SCD aged 8-80 years old; (2) individuals who were post bone marrow transplant for SCD; (3) parents/legal guardians/caregivers (including significant others, family, and friends) of individuals with SCD (newborn to 80 years) directly involved in decision making with/for that individual; (4) parents/legal guardians/caregivers (including significant others) of individuals who were post-bone marrow transplant for SCD who were directly involved in decision making with/for that individual; (5) stakeholders involved in any aspect of SCD; (6) health care providers who were directly involved in sickle cell health care, including but not limited to physicians, nurse practitioners, physician assistants, social workers, and nurses; and (7) those able to comprehend English.

We excluded family members/individuals/caregivers not directly involved in decision making regarding SCD health care, and stakeholders who were not involved in any aspect of SCD. We recruited participants in local, regional, and national SCD meetings as well as SCD clinics. We conducted semistructured qualitative interviews of stakeholders to elicit their experience in seeking information about, and making decisions related to, SCD and to identify decisional conflict (uncertainty), knowledge and expectations, values (what is important to patients), support and resources, decision types, timing, stages and learning, and personal clinical characteristics. We also explored what values were most important to patients to inform not only the development of the patient decision aid but also future approaches to therapy. The interviews were conducted utilizing both open- and closed-ended questions. Demographic and disease complication information were also collected from each participant. Interviews lasted 30-40 minutes and were transcribed verbatim. A coding scheme to organize data into themes was developed using QSR NVivo 10 software (QSR International Pty Ltd, Chadstone, Victoria, Australia). Each code was defined and sustained throughout the analysis, and these codes were eventually developed into categories. From the coding process, we retained the categories that we believed held the most explanatory power, and then the primary categories were further analyzed. In the initial steps of analysis, we gave all data equal consideration and we focused on variation across the data rather than frequency counts of concepts. Analysis concluded when we observed the replication of concepts. Ultimately, we developed categories from consistent patterns in the data. Thematic saturation was achieved when the team believed the development of categories addressed the research question. Inter-coder agreement was achieved in three steps: (1) one team member performed the initial coding of the transcripts, and as concepts developed, they were discussed and deliberated by the entire team; (2) once coding and deliberation was completed, a second team member coded all transcripts and verified the original coding scheme (in the event of disagreement between the two coders, a third team member served as arbitrator by also coding the transcripts in question); and (3) results were discussed until the entire team came to a consensus.

### Development of the Patient Decision Aid

The results of the needs assessment were used to create the storyboard for the patient decision aid, and through iterative cycles of refinement they were used in the creation of its first draft prototype. We developed the patient decision aid [41] in accordance with International Patient Decision Aids Standards (IPDAS) [42-44]. The patient decision aid was designed to describe SCD and provide information on all three treatment options, describe the positive and negative features of each option, and describe the likelihood of positive and negative outcomes. We used up-to-date scientific evidence, cited the sources in a reference and technical section, disclosed sources of funding, and disclosed any conflicts of interest. The patient decision aid was written at a Grade 5 equivalent reading level or less, and provided ways to help patients obtain additional information through means other than reading, such as audio and video. We also included patient stories to represent a range of positive and negative experiences.

### Field Testing of the Patient Decision Aid

Alpha testing for clarity, comprehensibility, and usability was completed by patients, stakeholders, and clinicians. The results of the alpha testing then informed the finalization of the prototype of the Web-based patient decision aid. We conducted two iterative cycles of beta testing with patients, caregivers, stakeholders, and health care providers from around the United States.

Qualitative interviews with patients and health care providers were conducted during the beta testing of the patient decision aid to elicit stakeholder perspective on the quality, accuracy of, and satisfaction with the content and presentation of the patient decision aid, as well as suggestions on how to improve it. After the first round of beta testing, all the qualitative interviews were contemporaneously analyzed, and when thematic saturation was achieved they were used to modify the prototype patient decision aid. This was then subjected to a second iterative cycle of beta testing. We then synthesized these findings and applied them to the design of the final patient decision aid.

We included all potential subgroups (ie, adults, adolescents, parents of adolescents, and parents of young children), and we focused our efforts on recruiting the largest number of patients possible to include individuals drawn from different demographic descriptions and who were considering different treatment options. While the recommended sample size in qualitative interviews is 12-90 individuals with a median of 30 [45], we planned a larger sample size to enable us to characterize patients based on their experiences with the intervention or usual care, as well as age and role (patient versus parent), while enabling us to achieve thematic saturation [45]**.**

### Benchmarking of the Patient Decision Aid

For benchmarking the final patient decision aid, we used the International Patient Decision Aids Standards self-evaluation instrument (IPDASi), a validated, interrater-reliable [46-49,50]. The IPDASi provided an internationally accepted benchmark to assess the quality of development, the process, the content, any potential biases, and the methods of field testing and evaluating a patient decision aid.

### Randomized Clinical Trial of the Efficacy of the Patient Decision Aid

We further evaluated acceptability and usability of this final patient decision aid among participants in a randomized clinical trial (NCT03224429). Inclusion and exclusion criteria are listed in Textbox 1.

**Inclusion and exclusion criteria of the randomized clinical trial.**

Inclusion criteria:

- Individuals with sickle cell disease (SCD) aged 8-80 years old

OR

- Parent/legal guardian of patients (age<18 years) with SCD who are directly involved in decision making regarding SCD health care treatment

OR

- Health care provider directly involved in care of individuals with SCD, including child of parent/legal guardian enrolled in study
- Patients/parents/caregivers who have made a past decision to not obtain treatment of the considered option or who have not obtained treatment of the chosen option in past 12 months
- Able to comprehend English
- Patients/parent/legal guardian who will have access to the internet from iPad, smart phone, or personal computer
- Patients <18 years may participate in Testing of Decision Aid, Cohort B, if they have participated in the Qualitative Needs Assessment first

Exclusion criteria:

- Family members/individuals/caregivers not directly involved in decision making regarding SCD health care
- Patient/parent/legal guardian who has already decided to begin and has started the treatment option
- Parent/legal guardian of child who is participating in Cohort B of this study
- Child <18 years of age of parent/legal guardian who is participating in Cohort A of this study and randomized to the control arm and not the decision aid arm
- Spouse, significant other, or other family member involved in decision making for child <18 years if parent/legal guardian of child already enrolled into this study.

Participants considering disease-modifying therapies for SCD were randomized to receive either the patient decision aid or standard care (SC) arms prior to deciding. Cohort A consisted of three subgroups: (1) parents or legal guardians of children aged <18 years old; (2) individuals with SCD who were between 18-28 years old; and (3) individuals with SCD who were >28 years old. Within each of these groups, we identified what particular treatment decision participants were considering (HU/BMT/CTT), and then assigned them to the HU, BMT, or CTT strata according to the intervention that they were contemplating. Cohort A subgroup participants were randomized to patient decision aid versus SC. SC was defined as usual care with a current health care provider in usual practice without the use of a patient decision aid. We collected demographic information from adults with SCD, or caregivers of pediatric patients, that included age, gender, race, education, marital status, and employment.

Participants in the patient decision aid arm completed surveys at randomization and were provided access to the patient decision aid with a unique access ID and password for the purpose of this study. Participants were scheduled for their second research visit to coincide with the completion of discussions with the health care provider/team regarding treatment options. Participants were asked to complete the study surveys via a paper version or online within 2 weeks of the office/clinic visit. Self-reported information regarding themselves or the patient they cared for was updated relative to SCD management and complications, since Consent/Visit 1 Participants received a monthly follow-up via telephone or email to verify their ability to access and navigate the website. Soon after, and within 4 months following their discussion with their health care provider, participants were scheduled for their final research visit. Participants were asked to complete the study surveys as either a paper or online version within 2 weeks of this office/clinic visit.

After completion of the final study visits and surveys and data collection on the patient decision aid arm, participants in the SC arm were offered password-protected access to the patient decision aid website. Participants were given 4 weeks to review this site, at which time they were asked to complete a series of questionnaires either electronically or via a paper version.

Adolescent patients aged 10-18 years old were assigned to cohort B and were offered the ability to view the patient decision aid without randomization. The purpose of cohort B was to test the quality and comprehension of information and the impact on daily life relating to management of SCD in patients <18 years of age.

### Outcomes Studied in the Clinical Trial

#### Overview

We used the measurement tools developed to operationalize the variables in the Ottawa decisional framework to study several patient centered outcomes.

#### Acceptability of the Patient Decision Aid

Acceptability of the patient decision aid was tested using the Acceptability of Education Survey (8 multiple choice questions, 2 short answer questions), [48] a validated measure of the comprehensibility of components of a patient decision aid, as well as its length, pace, amount of information, balance in presentation of information about options, and overall suitability for decision making [49-52].

#### Patient Knowledge and Understanding of Treatment Risks and Benefits

The impact on patient knowledge was tested by using a knowledge questionnaire. Since knowledge is different from understanding, we designed questions that tested understanding (specifically health literacy).

#### Patient Attitudes Towards Decision Making

The desirability or personal importance a participant assigns to the risks and benefits of a treatment option was determined by administering a Values survey (14 multiple choice questions, six fill in the blank questions) [48,52-54]. The measures of the Decision/Choice Predisposition scale (one multiple choice questions and four fill in the blank questions) [55] were used to determine if the participant was leaning towards, or had a propensity to select, an option. The Stage of Decision Making refers to the individual’s readiness to engage in decision making, progress in making a choice, and receptivity to considering or reconsidering an option. For the Stage of Decision Making survey (10 multiple choice questions) [56], participants indicated their stage of decision making with responses ranging from “I haven’t thought about the decision” to “I have made my decision and am unlikely to change my mind”. Decision support is most likely to be useful in individuals who are in active contemplation or are willing to consider or reconsider an option. The tool is not scored but it is used to screen out patients or to study the covariation in decisional conflict or interventions.

#### Impact on Decision Making

The impact of the patient decision aid on the decision-making process and on the treatment decision includes the preparation for decision making, the specific decision made, satisfaction with the decision-making process, and satisfaction with the decision. We used the Preparation for Decision Making scale (10 multiple choice items) [57] to assess a patient's perception of how useful a patient decision aid or other decision support intervention is in preparing the respondent to communicate with their practitioner at a consultation focused on making a health decision. It is not specific or temporally-related to a particular visit to the doctor and as such was well suited to this study.

#### Psychological Impact of the Patient Decision Aid on Decision Making

This was assessed by the decisional regret scale [58], which measures distress or remorse after a health care decision, the Decision Self-Efficacy Scale (11 multiple choice questions) [59], which measures self-confidence or belief in one’s ability to make decisions and includes participating in shared decision making, and the Decisional Conflict Scale (16 multiple choice questions) [60], which measures the uncertainty in choosing options and the modifiable factors contributing to this uncertainty.

#### Patient and Caregiver Perspectives Elicited by Qualitative Research Methods

Qualitative interviews were conducted at baseline for all cohorts, with a focus on how people prefer to learn about SCD, the information content (ie, what physicians and other health care personnel share with patients and caregivers), and medical decision making. Participants were asked about their questions for their health care providers and what they would like to learn about SCD. Qualitative interviews were then conducted within 4 weeks of visit 3 to evaluate the extent to which the method of educational tool (standard practice or patient decision aid) helped the participant to recognize that a decision needed to be made regarding treatment of SCD, to understand the values that affected the decision, and understand if and how these values were addressed with the health care provider in making the health care decision. Qualitative interviews of pediatric patients were conducted after they had accessed the patient decision aid in order to assess their involvement in decision making, their preferences regarding Web-based educational content, and their views about the quality and acceptability of the patient decision aid.

### Analytic Methods

We assumed that 30% of patients or parents participate in decision making with standard care, and that the rate of patient or parent participation would double with the intervention. Based on a 90% power and a 0.05 significance level, we required 110 patients, 55 in each group, for comparing 2 binomial proportions. To account for attrition we recruited 120 patients, 60 in each treatment group and 20 in each age group. Reasons for missing data were considered in the analysis. We also assessed the plausibility of the assumptions associated with the approach.

Systematic efforts were instituted to maximize follow-up. A calendar was maintained for the timing for subject procedures, and participants were contacted by telephone and email for reminders and scheduling. If a subject did not respond after five contact attempts, the subject was considered to be lost to follow up. We assumed that the data were missing at random and conducted simple imputation to calculate total scores and subscores. We then compared changes in these scores between the patient decision aid and SC control groups. We did not use model-based methods such as multiple imputation, as the purpose of this study was not to estimate a model but to evaluate the assigned intervention based on survey results over time. We then imputed the missing responses with the mean. We realize that imputing the mean preserves the mean of the observed data and these estimates remain unbiased, but we also realized that imputation with the means results in underestimation of the SE and introduces a source of bias.

## Results

### Decisional Needs Assessment

A total of 223 individuals, including adult patients, caregivers of pediatric patients, health care providers and other stakeholders, participated in qualitative interviews regarding decisional needs assessment about decision making for treatments for sickle cell disease (Table 1).

The participants had a higher level of academic achievement, with 86% reporting having had some college education. Thus, their views may or may not be representative of the sickle cell community at large. It should be noted that participants had predominantly been enrolled in local, regional and national conferences about SCD and so may have been more active about their own care. We included qualitative interviews conducted on entry into the randomized clinical trial in this analysis because patients had not been exposed to the patient decision aid yet.

**Table 1:** Subject flow and baseline demographics for the decisional needs assessment.

Demographic	Adult patient (n=63)	Caregiver (n=61)	Stakeholder (n=42)	Health care provider (n=56)
Age (years), median (range)	27 (18-66)	39 (16-71)	—^a^	—
Gender (female), n (%)	50 (79)	47 (77)	24 (57)	28 (50)
Race (African-American), n (%)	60 (96)	56 (92)	24 (57)	—
Ethnicity (non-Hispanic), n (%)	62 (98)	60 (98)	41 (98)	—
Education (some college or greater), n (%)	54 (86)	40 (65)	42 (100)	—
Employment (part or full time), n (%)	38 (60)	38 (63)	—	—
Married, n (%)	8 (12)	19 (31)	—	—

^a^Not applicable.

Qualitative analysis yielded the following major themes in the various aspects of decision making: how people prefer to learn about SCD, their thoughts about the content, and how they used this information for medical decision making. They discussed the content of the information shared by physicians and other health care personnel with patients and caregivers regarding what they would like to learn about SCD and what outcomes were important to them. While patients and caregivers relied on their conversations with their physicians for making decisions regarding treatment, they also shared several perspectives on how they would like to receive information that would help them participate in decision making (Textbox 2).

**Stakeholder preferences regarding Web-based educational resources to aid in decision making regarding treatment options.**

- High quality, unbiased, evidence-based information to guide decision making
- Interactive design with easily comprehensible, customizable content presented in a visually pleasing, user-friendly format with limited text and attractive graphics
- Hear directly from individuals like themselves regarding their experiences with treatments and treatment decisions
- Patient video testimonials on the participants covered
- Accurate and unbiased information about pros, cons, and outcomes of each treatment option

**Figure 1:**
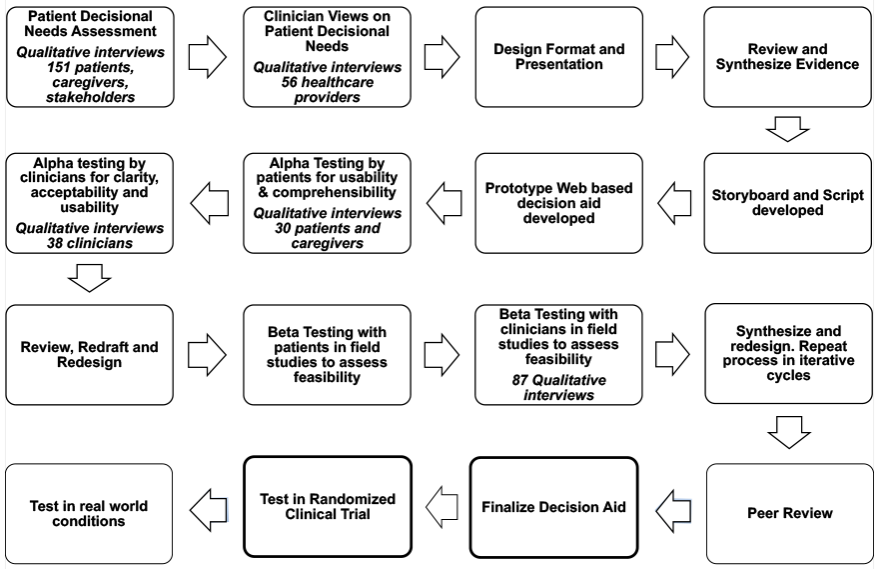
The iterative process for the development and testing of the patient decision aid using the Ottawa decisional framework.

In accordance with the stakeholder preferences, we designed the presentation format, synthesized evidence generated, created a script and storyboard, and drafted a Web-based prototype of the patient decision aid. The iterative process utilized for the development and testing of the patient decision aid using the Ottawa decisional framework is outlined in Figure 1. The following key elements were included in the design of the patient decision aid:

- Each line of text was designed to read at ≤5th grade level and tested using the Smog Readability Formula. Where necessary, definitions of words were provided.
- Minimizing of text and maximizing the use of graphics.
- High quality information which was subjected to extensive peer review by national SCD experts.
- Links to peer-reviewed references and credible sources of information.
- Information was divided into sections (sickle cell care, treatment options, values clarification, communication with physicians, etc).
- Content was displayed in accordion format to eliminate clutter and allow users to customize specificity and detail as desired.
- Ability to personalize content in the context of personal health information and a personalized folder with saved content, graphics, and videos.
- Values clarification exercises, with side by side comparison of treatment options based on values or combination of values.
- Ability to save information and values clarification exercises in preparation for discussions with their physician.

### Field Testing

Alpha testing for clarity, comprehensibility, and usability was completed with 30 patients/stakeholders and 36 clinicians. The results of alpha testing helped with the finalization of the prototype Web-based patient decision aid. We then conducted two iterative cycles of beta testing with 87 patients, caregivers, stakeholders, and health care providers from around the US. The qualitative interviews conducted as a part of the beta testing were also subjected to rigorous qualitative analyses.

The following themes emerged in the qualitative analyses of patient and health care provider interview transcripts from beta testing of the patient decision aid:

- A high level of satisfaction with the accuracy and quality of the content and presentation.
- Acceptability of the patient decision aid for ease of use, comprehensibility, and use of graphics.
- High perceived utility of the values clarification exercises.
- Satisfaction with the large number of video testimonials from patients and stakeholders sharing their personal narrative about medical decisions making regarding disease modifying therapy for SCD.

### Benchmarking the Patient Decision Aid

We evaluated the quality of the development process, content, potential biases, and the methods of field testing and evaluating a patient decision aid using the 12 domains from the 2006 IPDASi checklist. The patient decision aid met 61/62 standards (see Multimedia Appendix 1). However, since we were unable to provide stories of patients who had an adverse outcome after BMT in the patient decision aid, it did not meet all requirements.

### Randomized Clinical Trial

**Figure 2:**
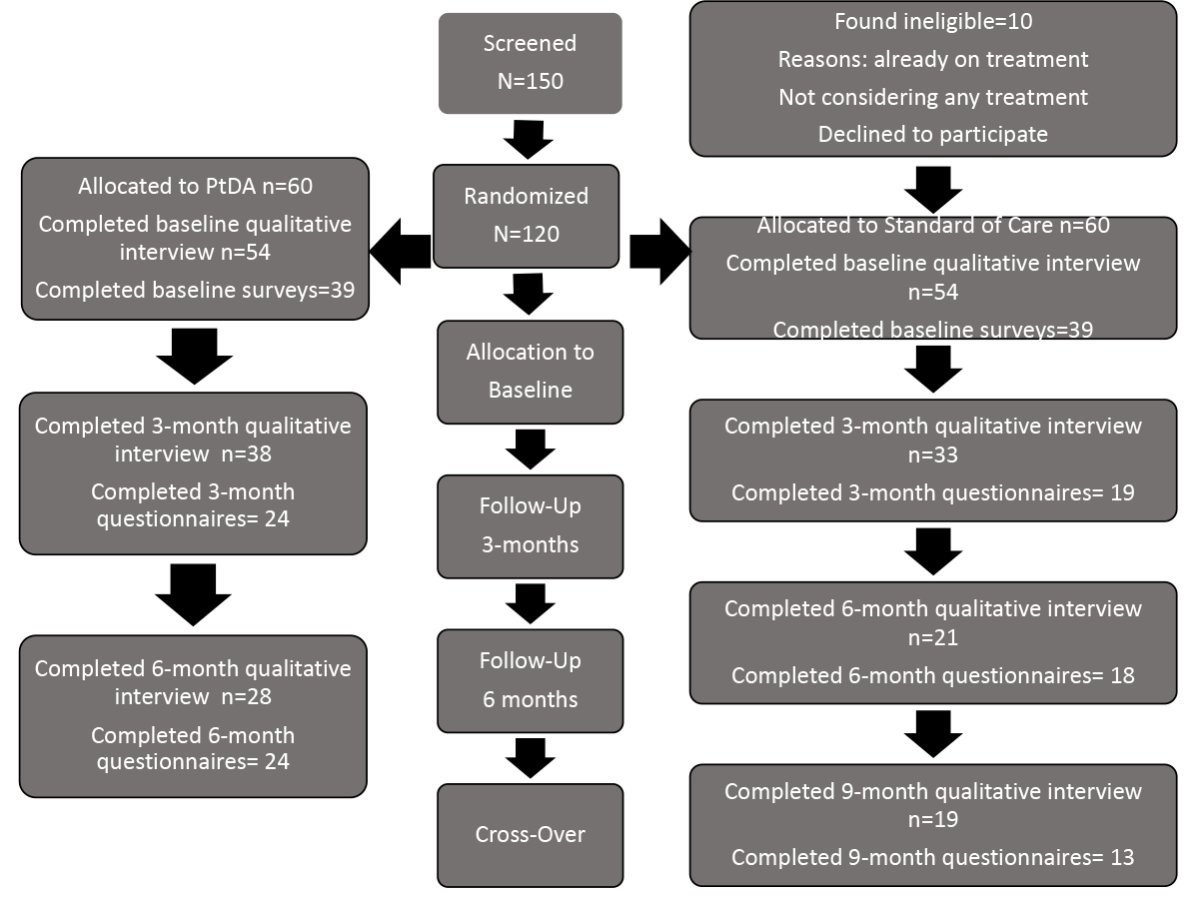
Consort diagram describing the recruitment in the randomized clinical trial. PtDA: patient decision aid.

We randomized 120 participants to a clinical trial of the patient decision aid to determine its impact on knowledge and decisional conflict, preparedness, and regret. Participants were assigned to HU, BMT, and CTT strata according to the intervention that they were contemplating (BMT 73, HU 29, CTT 18). Participants randomized to the patient decision aid underwent interviews and completed surveys at study assignment, then again three months later after having a chance to review the instrument, and then again after six months when they were likely to have made their therapeutic decisions. Participants assigned to the control arm provided the same measures at baseline and at three and six months. After six months, they were given access to the patient decision aid and were interviewed and completed surveys at seven months. Of all the participants in the trial, 76% (91/120) were female with a median age of 34 years (mean 35.5), 75% (90/120) were African American, 8% (11/120) were Hispanic, 80% (96/120) had some college education, 53% (64/120) were employed, and 26% (31/120) were married (Figure 2 and Table 2). Over the different stages of the study, 76 patients were lost to follow up. Of those patients lost to follow up, 72% (55/76) were female with a median age of 31.5 years (mean 33), 75% (57/76) were African American, 8% (6/76) were Hispanic, 72% (55/76) had some college education, 56% (43/76) were employed, and 21% (16/76) were married. Of the 120 patients enrolled, 16% (19/120) did not complete any study procedures and were lost to follow up despite our *a priori* defined methods to retain contact. Completion rates for different surveys, overall and at different timepoints, ranged from 43-68%. In a univariate analysis the age, gender, race, ethnicity, educational level, employment status, and marital status did not statistically significantly predict loss to follow-up (Table 2).

Of 120 patients enrolled in the study, completion rate of interviews varied between qualitative interviews and survey instruments and at different stages of the study. Qualitative interviews and surveys were completed by 101 and 78 patients at baseline, 71 and 48 patients at 3 months, 49 and 42 patients at 6 months, and 18 and 13 subjects at 9 months. These data suggest at least in this study the burden of completion of qualitative interviews may have been less than the completion of surveys and that participants seemed to lose interest in the study over time.

**Table 2:** Subject flow and self-identified demographics of all participants versus those who were lost to follow up.

Demographic	Lost to follow up (n=76)	Total participants (N=120)	*P* value
Age (years), median	31.5	34	.21
Gender (female), n (%)	55 (72)	91 (76)	.45
Race (African-American), n (%)	57 (75)	90 (75)	.99
Ethnicity (non-Hispanic), n (%)	69 (91)	109 (91)	.99
Education (some college or greater), n (%)	55 (72)	96 (80)	.19
Employment (part or full time), n (%)	43 (56)	64 (53)	.68
Married, n (%)	16 (21)	31 (26)	.42

### Acceptability Survey

Overall, 144 out of a maximum possible 300 individual surveys (response rate 48%) were completed on the acceptability of the patient decision aid information on SCD treatment options pertinent to BMT, CTT, or HU at different timepoints. A range of 58-85% (median 72%) of participants rated the information as good or excellent on various sections of the patient decision aid. Detailed descriptions of the response to each individual section is described in Table 3. In completing acceptability surveys, 106 participants also provided additional narrative comments. Participants said the website was informative, helpful, easy to understand, provided a lot of new information in one place, and was very helpful in making decisions. Participants appreciated being able to save information, to clarify values, and to view video testimonials.

**Table 3:** Results of the acceptability survey.

Criterion		Median (% ranking as good or excellent)
**Comprehensibility of different information sections (1=Poor, 2=Fair, 3=Good, 4=Excellent)**		
	Impact of sickle cell	3 (84)
	Risk factors	3 (72)
	Research	3 (57)
	Treatment options	3 (69)
	Evidence supporting self-care	3 (68)
	HU^a^/BMT^b^/CTT^c^	3 (68)
	Evidence about HU/BMT/CTT	3 (64)
	Stories about others	3 (58)
Amount of time learning took: (1=too much, 2=too little, 3=just right)		3 (72)
The amount of information was: (1=Poor, 2=Fair, 3=Good, 4=Excellent)		3 (72)
Found the information: (1=slanted towards self-care, 2=slanted towards interventions, 3=balanced)		3 (62)
Information was useful when making decision regarding HU/BMT/CTT (1=Yes, 2=No)		1 (87)
Ways to calculate risk factors (1=Easy, 2=Difficult)		1 (68)
Health history worksheet made the decision: (1=Easy, 2=difficult)		1 (81)
Portfolio worksheet made your discussion with the physician more: (1=Easy, 2=Difficult)		1 (81)
Did it provide information to help someone decide on whether to accept HU/BMT/CTT (1=Yes, 2=No)		1 (76)

^a^HU: hydroxyurea.

^b^BMT: bone marrow transplantation.

^c^CTT: chronic transfusion therapy.

### Values Survey

Patients/caregivers also completed validated values questionnaires [52] at multiple timepoints throughout the study, for a total of 172 out of a possible 420 completed surveys (43% completion rate). Intriguingly, at each timepoint in all subgroups, the median score for the values questionnaire (Table 4) was 11, the highest possible score. In the patient decision aid group, at the final research visit (visit three, at the six-month time point), 41/60 participants (68% completion rate) who completed the values survey all gave the highest possible score for the values queried, indicating that these values were their most important considerations for making decisions. At the final research visit, 33 participants also gave additional narrative comments following completion of the values questionnaire, with freedom from pain, not having to take medications, and improved quality of life among the most important considerations in considering a treatment. Being well informed about complications of a treatment and not being worse off because of complications were also major sources of caution.

**Table 4:** Values considered important by patients and caregivers regarding treatments.

Value	Median^a^ (25th, 75th percentile)
How important is it for you to know the complications of SCD^b^?	11 (11, 11)
How important is the benefit of reducing SCD-related complications?	11 (10, 11)
How important is the possibility of living longer due to a treatment?	11 (10, 11)
How important is risk of hair loss from hydroxyurea?	11 (7, 11)
How important is risk of darkening of nails due to hydroxyurea?	11 (7, 11)
How important is risk of cancer due to hydroxyurea?	11 (9, 11)
How important is the need for recurrent blood draws on treatment?	11 (8, 11)
How important is possibility of reducing stroke risk due to transfusion?	11 (10, 11)
How important is risk of transfusion reaction?	11 (10, 11)
How important is risk of infection due to transfusions?	11 (10, 11)
How important is risk of iron overload due to transfusion?	11 (10, 11)
How important is possibility of cure by BMT^c^?	11 (10, 11)
How important is risk of graft failure from BMT?	11 (10, 11)
How important is risk of graft versus host disease from BMT?	11 (10, 11)
How important is risk of infertility following BMT?	11 (5, 11)
How important is risk of death following BMT?	11 (11, 11)

^a^Evaluated on an 11-point Likert scale.

^b^SCD: sickle cell disease.

^c^BMT: bone marrow transplantation.

### Knowledge Survey

Participants completed a knowledge survey about SCD and treatment options, first at baseline and then at different time points (Table 5). Differences in the proportion of correct answers in the patient decision aid and in the SC control arm at all time points did not reach statistical significance. The study was powered based on differences detected in the group, since attempting to detect differences in impact on individual decisions in each group would have required an unrealistically large sample size. Patients were nonrandomly assigned to BMT, HU, or CTT subgroups based on the decision they were considering. Surprisingly, most of the participants indicated that they were considering the BMT option. Subgroup analysis was underpowered and not realistic given the low rate of compliance with survey completion, with further attrition over time. Thus, the small numbers and asymmetric distribution limited the numbers available for meaningful subgroup analysis. In addition, participants were given access to the entire patient decision aid containing information on all the treatment options, which could have resulted in them simultaneously considering more than one of the options available to them. We therefore believed that it was difficult to clearly separate the impact of the patient decision aid on different decisions, so we combined the three cohorts for comparison between the patient decision aid and SC control arms. Since there was no statistically significant difference in the knowledge scores between the patient decision aid and SC groups at any time point, we did not carry out analyses of comparison of difference in change in scores from baseline.

**Table 5:** Percentage of correct answers for knowledge questionnaire.

Time-point	Patient decision aid, N (Mean)	Standard care control, N (Mean)	*P* value
Baseline	39 (49.83)	38 (49.87)	.97
3 months	23 (52.90)	19 (52.90)	.12
6 months	22 (55.54)	17 (46.49)	.12
9 months (cross-over)	—^a^	12 (51.18)	—

^a^Not applicable.

### Choice Predisposition Survey

A total of 170 choice predisposition scale surveys (40.5% response rate) were completed by participants at different time points to indicate which treatment they were leaning towards. There was no statistically significant change in the predisposition scale following the use of the patient decision aid. The median scale score was 52 at baseline and 72 at 6 months *(*P*=*.57). Of 30 participants who completed the choice predisposition survey at six months, 7 indicated an inclination towards making a decision (23%). A total of 109 participants gave narrative comments on their choice predisposition survey. The major reasons for not making a choice for a treatment were: (1) a desire to think more about the decision; (2) not being convinced that the benefits outweighed the risks; (3) not being convinced that their disease was severe enough; and (4) not being convinced that the treatment would benefit them in particular. Those choosing a treatment did so: (1) to have a better quality of life; (2) to be free of pain; and (3) because they were confident they were making an informed decision.

### Stages of Decision Making

We scored stages of decision making on a six-point Likert scale ranging from, “I haven’t thought about the decision,” to “I have made my decision and am unlikely to change my mind.” The tool is not typically scored but is used to screen out patients or to study the covariation in decisional conflict or interventions. Differences in the median stages of decision making at baseline and at three and six months in the two arms were not statistically significant.

### Preparedness for Decision Making Survey

Individual subscales on a five-point Likert scale (ranging from not at all to a great deal) are summed and converted into a composite score, with a higher score indicating a greater preparedness for decision making. There were 74 participants (37 patient decision aid, 37 SC) with baseline data, 42 participants (23 patient decision aid, 19 SC) with three-month data, and 39 participants (22 patient decision aid, 17 SC) with six-month data. Of these, 38 had both three-month and baseline data, and 32 had both six-month and baseline data. Of the 10 survey questions, five had missing data. Four of those questions had only one missing value; one question had two. Again, we performed single mean imputation. There were no differences observed after three months among our 38 participants, but an overall difference at six months. At six months, the patient decision aid participants had a significantly higher change in preparedness for their decision-making score (*P*<.001) than the SC participants.

The mean diference of change in the preparedness for the decision making survey was not statistically significant at 3 months (*P*=0.16), but was statistically significant at 6 months *(P*<.001)

### Decisional Regret Survey

The 7 participants who made a treatment decision regarding HU and the 4 patients who made a treatment decision regarding CTT in the course of the study completed the decisional regret scale [58]. No patient in the subgroup considering a decision regarding BMT completed a decisional regret survey. All individuals indicated a low level of regret following the HU or CTT decision, with scores ranging from 20-25 on a 100-point scale, but the number of individuals who made a decision was not large enough for any formal statistical analysis. Follow-up qualitative interviews after the completion of the study with the 46 participants in the patient decision aid group and the 16 participants in the SC group who crossed over to the patient decision aid group at the end of the study indicated that participants were able to carefully consider pros and cons, clarify their own values, and felt empowered to make a decision about a therapeutic option.

### Decision Self-Efficacy Survey

Regarding the decision self-efficacy survey, there were 78 participants (39 patient decision aid, 39 SC) with baseline data, 42 participants with three-month data, and 42 participants (23 patient decision aid, 18 SC) with six-month data. Of these, 36 had both three-month and baseline data and 35 had both six-month and baseline data. Of the 11 survey questions, 4 had missing data. Three of those questions had only one missing value; one question had two. Again, we performed single mean imputation for partially completed surveys. There was a statistically significant difference in the change in the decisional self-efficacy score (*P*=.05) observed after three months among our 36 participants. However, the difference in the change in decisional self-efficacy scores from baseline among the 35 participants with baseline and six-month data was not statistically significant, though the patient decision aid participants had a greater change in self-efficacy score (*P*=.06) than the SC patients.

### Decisional Conflict Survey

During the randomized clinical trial, 172 decisional conflict scales were completed. Decisional conflict scale responses were scored for the total score, uncertainty subscore, informed subscore, values clarity subscore, support subscore, and effective decision subscore. The decisional conflict scale consists of 16 items in 5 response categories and is scored on a 0-100 scale.

There were 41 participants that completed both the baseline and the three-month surveys. Analyses were conducted with two-tailed *t* tests (alpha=.05) as hypotheses were based on inequality rather than specifying the direction of the inequality. A total of 9 participants had some missing data, with only one participant having missed more than one question. We do not believe that certain treatment preference subgroups were more likely to have missing values, as 7 were interested in BMT and 2 in HU *(P>*.99 by Fisher’s exact test). Similarly, 4/9 participants were randomly assigned to the patient decision aid group and 5/9 to the SC group. Certain questions did not seem to be more likely to be missing than others, as the number of missing questions ranged from 0-3 for each of the 16 questions. However, we confirmed the results of the *t* test with a nonparametric method that didn’t utilize SE. Table 6 depicts the total scores and subscale scores difference over time, with the mean difference representing the baseline scores minus the 3-month scores. The *P* value refers to the difference in the mean differences from baseline. We tested whether the average change in scores equaled zero, and in all cases, inference from the nonparametric Wilcoxon test matched the results of the *t* test (Table 6). Average difference and *P* value are shown in Table 6. Based on these analyses, the patient decision aid group had a significantly greater decrease in the informed subscore of decisional conflict when comparing the three-month datae to the baseline data. Of the 79 patients with baseline data and 42 patients with six-month data, there were 36 participants with results from both time points. The six-month versus baseline results are depicted in Table 7. There was no difference in total decision conflict score or subscale scores when comparing the six-month survey with baseline data. This lack of difference remained when we compared patients by random assignment.

**Table 6:** Comparison of change in decision conflict scale from baseline at the 3-month time point for the patient decision aid group versus the SC group.

Parameters		Mean difference, patient decision aid	Mean difference, SC^a^	*P* value
**Subscales**				
	Uncertainty	5.21	1.32	.38
	Informed	–14.65	1.75	.003
	Values clarity	–4.17	–4.39	.97
	Support	1.14	–7.02	.15
	Effective decision	1.69	–3.46	.35
Total score		–1.91	–2.43	.81

^a^SC: standard care.

**Table 7:** Comparison of change in decisional conflict scale from baseline at the 6-month time-point according to randomization to Patient decision aid versus SC.

Parameters		Mean difference, patient decision aid	Mean difference, SC^a^	*P* value
**Subscales**				
	Uncertainty	10.1	–1.2	.34
	Informed	–2.4	–6.9	.72
	Values clarity	–2.4	–4.2	.89
	Support	5.2	–6.0	.40
	Effective decision	6.8	–1.7	.48
Total score		3.7	–3.8	.52

^a^SC: standard care.

### Qualitative Interviews After Use of the Patient Decision Aid in the Randomized Clinical Trial

Another set of qualitative interviews was conducted within three months of the initial education about/use of the patient decision aid. These qualitative interviews provide substantial insight into the user experience of the patient decision aid and as such are reported here in some detail. The following were the major themes that emerged from these interviews. Users of the patient decision aid:

- Overwhelmingly endorsed and appreciated the patient decision aid and found it to be very educational for decision making;
- Reported that the information was concise and presented well;
- Particularly noted that good information was provided on risks and benefits;
- Noted that doctors did not elaborate as much as patients wanted them to on the risks of treatment (especially HU) and that the patient decision aid was a useful supplementary source of information;
- Thought that the testimonials were very important and had an impact on decision making, helping users see patients who had experience with the treatment;
- Felt that the identification of risks, benefits, family needs, and what is important through questions and information provided in the steps (values clarification) helped them to decide whether to pursue the treatment;
- Felt that information on the website empowered users to talk to doctors because it helped to identify information patients/parents should ask about and gave them a place to start when bringing up a treatment with the doctor;
- Particularly felt that they did not receive information about BMT as a treatment option from their health care providers and that information was provided in the patient decision aid. Patients/parents reported that when they brought up BMT they were sometimes told they did not qualify or the doctor would not recommend that treatment without sending them to a specialist;
- Felt that not all patients/parents brought up treatments to the doctors but went about trying to find information on their own. These methods of learning included the internet, talking to others, and conferences. When patients/parents talked with doctors about a treatment they felt that the doctors did not always give enough information regarding risks and benefits and that the information did not include patient experiences, which was deemed to be very important.

### Qualitative Interviews of Pediatric Patients (Cohort B) After Using the Patient Decision Aid

We enrolled 16 adolescents in cohort B, which was open to adolescents under the age of 18, and they were nonrandomly assigned to the patient decision aid arm. Participants completed a baseline decisional needs qualitative interview and were then given the opportunity to view the patient decision aid. During follow-up, the patients completed an interview about their experience with viewing the patient decision aid and they were also offered an opportunity to take the self-efficacy scale, but none did. Qualitative analysis was performed on the transcripts of the 29 interviews (16 at baseline, 6 at 3 months, and 7 at 6 months). The following themes emerged from the baseline interviews: (1) patients reported being aware of information about self-management and supportive medical treatments; (2) they reported being involved along with their parent in the medical decision-making process; (3) they reported an eagerness to learn from high quality sources on the internet; (4) they mainly accessed the internet over the telephone; and (5) they indicated a desire to see information that was in graphic form rather than as text. In follow-up interviews, patients reported that they found the patient decision aid to be easy to use, easy to read and understand, and that it helped them speak with their physicians.

### Analysis of Online Usage of the Patient Decision Aid

We analyzed patterns of use for the patient decision aid with the online Google Analytics tool from the opening of the website to the general public following the completion of the clinical trial on October 1, 2016. From then to March 17, 2019 there have been 38,787 page views by 4587 users from 103 countries, with the United States being responsible for 55.73% of the sessions. The contents of the site were accessed in US English by 3281 users (71.39%). The bounce rate (ie, the proportion of single page sessions of zero second duration with no interaction) was 52%. The average session lasted 7.27 minutes and covered 4.72 pages. The 2017 content marketing benchmark report analyzed Google analytics data from 181 websites from 2015-2016 in order to establish industry benchmarks across a wide swath of web analytics metrics [61]. Compared with the industry benchmarks for bounce rate (53-67%), session duration (1:47-3:38 minutes), and pages viewed per session (1-2 pages), the patient decision aid performed very well. While the site has been accessed in 43 states and 92 metro areas in the United States, more than 70% of the sessions have occurred in 10 states. The site was accessed using a mobile device in 1/3 of the sessions and 77% of the users were in the 18-54 years old age group. The patient decision aid’s Facebook page, which is meant to promote the site, has received over 100 likes.

## Discussion

### Primary Findings

In this paper, we reported the development, completion, and testing of the first ever patient patient decision aid for SCD [41], which was created with the extensive engagement of patients, their caregivers, health care providers, community advocates, and policy makers. Using a theory-based, iterative process based on the Ottawa decision support framework and large-scale community engagement, we obtained substantial input from these stakeholders on the conception, design, development, alpha and beta testing, finalization, and peer review of the patient decision aid. Stakeholder input also yielded insights into how patients and caregivers navigate decision making for SCD [62,63].

Overall, both the qualitative interviews of participants obtained during the beta testing phase and during the randomized clinical trial) and the surveys of acceptability indicated a strong endorsement from patients and caregivers regarding the utility and ease of use of the patient decision aid. Traffic to the website, the proportion of visitors who left immediately, the proportion of new visitors, the amount of time spent by the average visitor, and the average number of pages visited per session all met and compared favorably with the industry standards for Web-based education [64-66]. The patient decision aid met all but one of 62 benchmarks for patient decision aids established by the IPDAS collaboration. Specifically, the patient decision aid did not include patient stories of those with an adverse outcome after BMT because no caregiver of a patient who had an adverse outcome of BMT came forward to share their story. In the patient decision aid arm, compared to the SC arm, there were statistically significant differences in improvement in decisional self-efficacy, a reduction in the informed subscore of the decisional conflict at three months, and improvement in preparedness for decision making at six months. In addition, there were no statistically significant differences in change in knowledge or preparedness for decision making at three months, or other domains of decisional conflict or change in self efficacy at six months. However, these results must also be viewed against the overall high dropout rate in survey completion in the randomized controlled trial. We were unable to draw conclusions about the decision quality (ie, whether or not people made decisions congruent with their values) for two reasons, one of which was that the median score that participants assigned to each value queried was the highest possible score, suggesting that this study identified the most important values that patients and caregivers considered when making decisions. However, the remarkable homogeneity of the scores that this population assigned to each of these values made it impossible to study any value-based differences. Second, very few individuals completed the decision regret scale, and most of those who completed it were in the SC arm. Overall, the findings of this study suggest that the patient decision aid provided high-quality information and enabled patients and their caregivers to work with their physicians to make treatment decisions based on their own values and preferences.

Prior studies indicated that, as compared with usual care, the patient decision aid can better improve people's knowledge regarding therapeutic options and reduce their decisional conflict related to feeling uninformed and unclear about their personal values [67]. However, in this study we did not find any significant differences or improvement in knowledge, instead observing reduced decisional conflict, improved preparation, and improved self-efficacy for decision making. These observations are in keeping with the findings of systematic reviews of the utility of patient patient decision aids, which suggest that while a patient decision aid may improve knowledge, reduce decisional conflict, and stimulate patients to be more active in decision making, they have little effect on satisfaction and a variable effect on decisions [37-40].

Stakeholders representing the SCD community, a population dealing with a serious chronic illness and disproportionate socioeconomic disadvantage, indicated a strong preference for a Web-based educational resource and demonstrated the acceptability and usability of such an instrument. The patient decision aid provides detailed values clarification exercises in the section, “What is important for me.” This section allows users to clarify their own values by directly rating the importance of each attribute of a decision after they have viewed the information. Users can compare treatment options by placing predefined values as prioritized by them and then comparing pros and cons of each of the options side by side. While there are at least 98 different values clarification methods, there is a paucity of data on the optimal design of values clarification methods to be used [68,69]. The most promising design feature identified is explicitly showing people the implications of their values by, for example, displaying the extent to which each of their decision options aligns with what matters to them [68,69]. We have now included this as well as several other values clarification methods. Of note, during the qualitative interviews of participants, they overwhelmingly endorsed the perceived utility of the values clarification exercises. This study thus demonstrates the potential for use of a patient decision aid by other racial and minority ethnic groups dealing with chronic illness.

Our tests of efficacy were inconclusive, so more research is needed to determine how patients and caregivers use the patient decision aid in decision making and how the patient decision aid can be used in supporting SCD treatment decisions. While a patient decision aid is a decision support tool, patient and caregiver participation in shared decision making may be related to patient, physician, and decision characteristics, as well as socioeconomic and demographic factors [63,70]. This study did not involve an intervention aimed at influencing physician behavior regarding involving patients in decision making. It is possible that this may have limited the impact of the use of the patient decision aid on patient/caregiver involvement in decision making. Further studies are therefore required to determine how physician involvement in incorporating a patient decision aid in discussions with patients may contribute to shared decision making in real world settings. Crosby et al have described the use of patient decision aids for HU in SCD patients that included coaching by clinicians [71]. Further study is needed to develop and test coaching methods for use of the patient decision aid to determine if they will improve both its utilization and utility.

HU, CBT, and BMT have disparate therapeutic intents and side effects, and they may be offered at different stages of disease progression according to evidence-based guidelines rather than simultaneously. We nonrandomly assigned subjects to the study arm based on the decision they were considering, without attempting to verify whether the patient was eligible, the treatment was recommended or was being actively considered.

The study population was split in each arm into groups of 20, representative of each age group and the treatment option they were looking to decide on. As such, the numbers were too small to study the impact of the patient decision aid in each age group for each type of decision. Instead, we had to undertake pooled analyses of these types of decisions. We were intrigued by the fact that most patients wanted to learn about BMT, which was unanticipated because until now only a small number of patients have undergone this treatment. This choice may be reflective of several factors including increasing interest in, or the lack of information about, BMT. It may also be reflective of the fact that information about HU is generally available through other sources, but that there is a general lack of information about BMT. Since the pros and cons and intent of each treatment is different, future studies may consider examining the efficacy of a patient decision aid for individual age ranges and individual decision types.

A major limitation of this study is the large amount of missing survey completion data in the randomized control trial. This introduces a source of bias and limits our ability to draw statistically valid conclusions. The likely causes of missing data in the study are the burden of surveys to be completed, geographic dispersion of participants around the country, and the fact that investigators had no direct contact or ongoing therapeutic relationship with participants. This underscores the inherent difficulty in conducting studies with national enrollment through direct contact with patients in the absence of an ongoing therapeutic relationship with the investigators. We chose to recruit patients primarily in national conferences to access a large, nationally representative population who would provide their candid opinion about the decision-making process without concerns about offending a health care provider. However, this approach limited our ability to follow up with patients during routine clinical encounters. Participants in the study had unusually high levels of academic achievement, with 80% reporting at least some college education. Further, the majority were attending national or regional sickle cell conferences. It is thus possible that the participants were unusually active regarding their care and may not be representative of the SCD population at large. We anticipate that in studies basing their recruitment in sickle cell clinics, we are more likely to have access to the entire range of activity of the general sickle cell population. The evaluation measures used in this study are those that are included in the Ottawa decision framework. However, it is possible that health literacy may have contributed to the burden of completion of surveys, as there was a long interval between first access to the patient decision aid and the offering of surveys for completion. Future trials may also consider limiting subject burden of survey completion and planning a short interval between an intervention and assessment to be better able to test various aspects of efficacy of the patient patient decision aid in patients with SCD.

### Conclusions

We created a patient decision aid for patients with SCD with engagement and input from consumers, patients, caregivers, physicians, and other stakeholders in the conception, design, development, alpha and beta testing, finalization, peer review, and implementation of this resource. The development stage and qualitative interviews among trial participants demonstrated a high degree of acceptability of the patient decision aid among users. While there were statistically significant improvements in decisional conflict and preparation for decision making, because of the large amount of missing data in the completion of follow-up surveys we cannot draw conclusions about the effectiveness of the patient decision aid in improving patient involvement in decision making, and in decision-making quality.

## Appendix

Multimedia Appendix 1 Completed IPDAS checklist Indicating Compliance of sickleoptions.org with IPDAS standards.

Multimedia Appendix 2 CONSORT-EHEALTH checklist (V 1.6.1).

## Abbreviations

BMT: bone marrow transplantation
CTT: chronic transfusion therapy
HU: hydroxyurea
IPDAS: International Patient Decision Aids Standards
IPDASi: International Patient Decision Aids Standards self-evaluation instrument
SC: standard care
SCD: sickle cell disease
