# Supplementary material for: Comparative Effectiveness of a Web-Based Patient Decision Aid for Therapeutic Options for Sickle Cell Disease: Randomized Controlled Trial
Source: J Med Internet Res. 2019 Dec 4;21(12):e14462. doi: 10.2196/14462 (PMC6934048; doi:10.2196/14462)
Supplement: Multimedia Appendix 1 [file jmir_v21i12e14462_app1.pdf]

Appendix I: Completed IPDAS checklist Indicating Compliance of sickleoptions.org with IPDAS standards

International Patient Decision Aid Standards Collaboration

Criteria Checklist

This checklist was published in the following article:

Elwyn G, O'Connor A, Stacey D, Volk R, Edwards A, Coulter A, Thomson R, Barratt A, Barry M, Bernstein S, Butow P, Clarke A, Entwistle V, Feldman-Stewart, D, Holmes-Rovner M, Llewellyn-Thomas H, Moumjid N, Mulley A, Ruland C, Sepucha K, Sykes A, Whelan T. Developing a quality criteria framework for patient decision aids: online international Delphi consensus process. *BMJ* 2006;333:417. [http://www.bmj.com/cgi/content/full/333/7565/417]

I. **Content**

Provide **information** about options in sufficient detail for decision making?

	Yes	No
Does the patient decision aid describe the health condition?	X	
Does the patient decision aid list the options?	X	
Does the patient decision aid list the options of doing nothing?	X	
Does the patient decision aid describe the natural course without options?	X	
Does the patient decision aid describe procedures?	X	
Does the patient decision aid describe positive features [benefits]?	X	
Does the patient decision aid describe negative features of options [harms / side effects /	X	
Does the patient decision aid include chances of positive / negative outcomes?	X	
Does the patient decision aid describe what test is designed to measure?	X	
Does the patient decision aid include chances of true positive, true negative, false positive, false negative test results?	X	
Does the patient decision aid describe possible next steps based on test result?	X	
Does the patient decision aid include chances the disease is found with / without screening?	X	
Does the patient decision aid describe detection / treatment that would never have caused problems if one was not screened?	X	

Present **probabilities** of outcomes in an unbiased and understandable way?

	Yes	No

Does the patient decision aid use event rates specifying the population and time	X	
Does the patient decision aid compare outcome probabilities using the same denominator?	X	
Does the patient decision aid compare outcome probabilities using the time	X	
Does the patient decision aid compare outcome probabilities using the scale?	X	
Does the patient decision aid describe uncertainty around probabilities [words, numbers, diagrams]?	X	
Does the patient decision aid allow the patient to select a way of viewing probabilities based on their own situation [e.g. age]	X	
Does the patient decision aid place probabilities in context of other events?	X	
Does the patient decision aid use both positive and negative frames [e.g. showing both survival and death rates]	X	

Include methods for clarifying and expressing patients' values?

	Yes	No
Does the patient decision aid describe the procedures and outcomes to help patients imagine what it is like to experience their physical, emotional and	X	
Does the patient decision aid ask patients to consider which positive and negative features matter most?	X	
Does the patient decision aid suggest ways for patients to share what matters most with others?	X	

Include **structured guidance** in deliberation and communication?

Does the patient decision aid...	Yes	No
Does the patient decision aid provide steps to make a decision?	X	
Does the patient decision aid suggest ways to talk about the decision with a health professional?	X	
Does the patient decision aid include tools [worksheet, question list] to discuss options with others	X	

II. **Development Process**

Present information in a **balanced manner**?

	Yes	No
Is the patient decision aid able to compare positive / negative features of	X	
Does the patient decision aid show negative / positive features with equal detail [fonts, order, display if statistics]?	X	

Have a **systematic development process**?

	Yes	No
Does the patient decision aid include developers' credentials / qualifications?	X	
Does the patient decision aid find out what users [patients, practitioners] need to discuss options?	X	
Does the patient decision aid have a peer review by patient / professional experts not involved in development and field testing?	X	
Has the patient decision aid been field tested with users patients facing the	X	
Has the patient decision aid been field tested with practitioners presenting		
The field tests with users [patients, practitioners] show the patient decision aid is acceptable?	X	
The field tests with users [patients, practitioners] show the patient decision aid is balanced for undecided patients?	X	
The field tests with users [patients, practitioners] show the patient decision aid is understood by those with limited reading skills?	X	

Use Up to date **scientific evidence** that is cited in a reference section or technical document?

	Yes	No
Does the patient decision aid provide references to evidence used?	X	
Does the patient decision aid report steps to find, appraise, summarize	X	
Does the patient decision aid report date of last update?	X	
Does the patient decision aid report how often patient decision aid id updated?	X	
Does the patient decision aid describe quality of scientific evidence [including lack of evidence]?	X	
Does the patient decision aid use evidence from studies of patients similar to those of target audience?	X	

Disclose **conflicts of interest**?

	Yes	No
Does the patient decision aid report source of funding to develop and distribute the patient decision aid?	X	
Does the patient decision aid report whether authors or their affiliations stand to gain or lose by choices patients make after using the patient decision	X	

Use **plain language**?

	Yes	No
Is the patient decision aid written at a level that can be understood by the majority of patients in the target group?	X	
Is the patient decision aid written at a grade 9 or equivalent level or less according to readability score [SMOG or FRY]?	X	

Does the patient decision aid provide ways to help patients understand information other than reading [audio, video, in-person discussion]?	X	

Meet additional criteria if the patient decision aid is **internet based**?

	Yes	No
Does the patient decision aid provide a step-by-step way to move through the web pages?	X	
Does the patient decision aid allow to search by key words?	X	
Does the patient decision aid provide feedback on personal health information that is entered into the patient decision aid?	X	
Does the patient decision aid provide security for personal health information entered into the patient decision aid?	X	
Does the patient decision aid make it easy for patients to return to the patient decision aid after linking to other web pages?	X	
Does the patient decision aid provide security for personal health information entered into the patient decision aid?	X	
Does the patient decision aid permit printing as a single document?	X	

Meet additional criteria if **stories** are used in the patient decision aid?

	Yes	No
Does the patient decision aid use stories that represent a range of positive and negative experiences?		X
Does the patient decision aid report if there was a financial of other reason why patients decided to share their story?	X	
Does the patient decision aid state in an accessible document that the patient gave informed consent to use their stories?	X	

III. **Effectiveness:** Does the patient decision aid ensure decision making is informed and values based?

Decision process leading to **decision quality**…

	Yes	No
The patient decision aid helps patients to recognize a decision needs to be	X	
The patient decision aid helps patients to know options and their features?	X	
The patient decision aid helps patients to understand that values affect decision?	X	
The patient decision aid helps patients to be clear about option features that matter most?	X	
The patient decision aid helps patients to discuss values with their practitioner?	X	
The patient decision aid helps patients to become involved in preferred ways?	X	

A pdf version of this checklist is available for download on http://www.decisionlaboratory.com

Note: Based on this IPDAS checklist, a new instrument has been developed to assess the quality of decision support interventions – IPDASi. For details see: http://www.ipdasi.org/ or contact: IPDAS@Cardiff.ac.uk
